# The Potential of Anti-Diabetic Rākau Rongoā (Māori Herbal Medicine) to Treat Type 2 Diabetes Mellitus (T2DM) Mate Huka: A Review

**DOI:** 10.3389/fphar.2020.00935

**Published:** 2020-06-30

**Authors:** Jonni Hazeline Koia, Peter Shepherd

**Affiliations:** ^1^Division of Health, Engineering, Computers and Science, School of Science, University of Waikato, Hamilton, New Zealand; ^2^Department of Māori Health and Metabolic Disease, Te Reo Tipu Research Centre, Waikato, New Zealand; ^3^Department of Molecular Medicine, University of Auckland, Auckland, New Zealand

**Keywords:** type 2 diabetes mellitus, mate huka, anti-diabetic rākau rongoā, kūmarahou (*Pomaderris kumeraho A.Cunn*.), karamu (*Coprosma robusta Raoul*), kawakawa (*Macropiper excelsum G.Forst. Miq*.), mātauranga Māori, Te Reo Tipu Research

## Abstract

T2DM (type 2 diabetes mellitus, or Māori term “mate huka”) is a major long-term health issue in New Zealand particularly among the Māori community. Non-insulin drugs commonly used in New Zealand for the treatment of T2DM have limits to their efficacy as well as side effects, which are of concern for diabetics. As such, the potential for natural products such as traditional rākau rongoā are of interest for potentially preventing the development of T2DM or improving the treatment of the disease. In particular, anti-diabetic effects have been reported for rākau rongoā such as karamu, kūmarahou, and kawakawa. Natural products have been identified in karamu, kūmarahou, and kawakawa that have documented potential effects on glucose metabolism that could contribute to the anti-diabetic effect of these rākau rongoā. As such, this could provide scientific insight into the mātauranga (traditional knowledge) developed over generations by Māori. However, detailed laboratory based and clinical studies would be required to understand and validate these properties of karamu, kūmarahou, and kawakawa, and to understand how they can be used in T2DM treatment. Social determinants of indigenous health such as language, culture, traditional knowledge, and identity, are important in understanding the relationship Māori have with their land and the mātauranga they developed of the medicinal properties within their rākau rongoā, over many centuries. Interestingly, traditional Māori views towards scientific research using animal models to test rākau rongoā are varied but supportive. Furthermore, cultural issues surrounding Māori mana motuhake (self-determination) of traditional rongoā Māori healing practices and the inequity faced by many kairongoā (rongoā Māori practitioners) and tohunga (healers) compared to mainstream health are a current issue within the New Zealand health system. As such, a cultural holistic approach for T2DM care among Māori would be advantageous. This review will outline the available evidence supporting the anti-diabetic efficacy of karamu, kūmarahou, and kawakawa. Currently though there is a lack of molecular research to understand the mechanisms of this efficacy, as such this review will also outline Te Reo Tipu Research, a kaupapa Māori framework for molecular and genomic research on taonga flora.

## Introduction

T2DM is considered one of the fastest growing long-term health conditions in New Zealand. Diabetes has doubled in New Zealand from 125,000 to 250,000 over the past 10 years, where 90% (225,000) of these new diagnoses are T2DM (Diabetes New Zealand, 2019). For every person diagnosed with T2DM there is usually a family member or caregiver who also “lives with diabetes” in a support role. This means that an estimated 450,000 New Zealanders are affected by T2DM every day, which is approximately 10% of the New Zealand's population. The estimated direct cost of T2DM in New Zealand has been forecasted to rise from $1,310 million in 2016/17 to $1,770 million in 2021/22 (PricewaterhouseCoopers, 2008).

The prevalence of T2DM “mate huka” among Māori is three times more when compared to other New Zealanders. The age of onset is also significantly earlier in Māori. The New Zealand Ministry of Health also confirms that T2DM prevalence among Māori children under the age of 15 years old is also increasing.

Diabetes is a chronic disease where the body is unable to maintain normal glucose homeostasis. Insulin released from beta cells in the Islets of Langerhans of the pancreas in response to high blood glucose levels act on liver, muscle, and fat cells to stimulate the uptake of glucose to be stored as glycogen or metabolized *via* glycolysis and oxidative phosphorylation.

The major metabolic defect of T2DM are; 1) insulin resistance where the sensitivity of insulin action in liver, muscle, and adipose tissue is impaired and 2) reduced ability of the pancreatic beta cells to produce enough insulin to compensate for insulin sensitivity loss.

Insulin resistance is a very complex process that can occur at different levels and paths of glucose transport (Gutierrez-Rodelo et al., 2017). Insulin resistance is usually associated with the development of an inflammatory state and can occur as a result of insulin modulation, deactivation of the insulin receptor complex, defect in the signal transduction modulations. The latter can involve activation of JNK-1 protein kinase preventing insulin responsive substrate one (IRS-1) being activated and also a change in the glucose transporter protein. Insulin resistance usually precedes and contributes to the development of pancreatic beta cell malfunction and as such, the reversal of insulin resistance is important in T2DM prevention and treatment

Historically the prevalence of diabetes among Māori was low prior to pre-European settlement and the adaptation to a western style diet and lifestyles is thought to have contributed towards high rates of diabetes among Māori. A case study has indeed confirmed T2DM can be improved by lifestyle changes with Māori reverting to traditional food intake and physical activity (Randell, 2010). Given this, culturally relevant approaches to T2DM prevention and care among Māori could prove beneficial, which highlights the importance of considering social determinants of health such as language, culture, history, and traditional knowledge, particularly mātauranga Māori relevant to rākau rongoā as a food and medicinal plant.

## Current T2DM Drug Treatments in New Zealand

T2DM is a progressive condition caused by the body gradually producing less and less insulin over time and while there is no cure for it, it can be managed through physical activity and healthy eating. Despite these efforts many will come to need drugs or insulin to manage their diabetes. Apart from insulin, the New Zealand health system currently provides five main classes of anti-diabetic drugs for T2DM patients.

The first line of drugs used to treat T2DM in New Zealand is the biguanide metformin. Early studies indicated that biguanides improve glycemia by acting on the liver *via* AMP activated protein kinase (AMPK)-dependent activation and AMPK-independent mechanisms *via* mitochondrial respiration inhibition (Foretz et al., 2019). Recent research has indicated that biguanides work in a much more complex way reflecting its multiple modes of action. Biguanides are also thought to act through mitochondrial glycerophosphate dehydrogenase inhibition, lysosomes and *via* the gastrointestinal microbiome. Furthermore, metformin is thought to target the gut (Bailey et al., 1994; Bailey et al., 2008), by increasing anaerobic glucose metabolism in enterocytes, which leads to low glucose uptake and high lactate in the liver. More recently it has also been shown that metformin stimulated release of GDF15 from gut cells showing its beneficial effects on energy balance and body weight (Coll et al., 2020). As such, the gut is now seen as a major target organ for metformin action, and that the liver may not be important for metformin action for those with T2DM as earlier thought (Rena et al., 2017). However, overall the mode of action for biguanides are still not fully understood.

The next line of therapy in New Zealand are sulphonylureas that work by stimulating the pancreas to produce more insulin. Sulphonylureas bind to the sulphonylurea receptor (SUR)‐1 subunit of pancreatic β‐cell ATP‐sensitive potassium (K_ATP_) channels, which leads to their closure and resulting membrane depolarization, therefore opening the voltage‐dependent calcium channels, causing intracellular calcium concentrations to increase, with a subsequent release of insulin (Douros et al., 2018). Sulphonylureas are marketed in New Zealand as gliclazide, glipizide, and glibenclamide.

Very recently, the DPP-4 inhibitor vildagliptin has become available in New Zealand for treating T2DM. This stops the normally rapid breakdown of glucagon-like peptide-1 (GLP-1) that is released from the intestine after eating. Its primary function is to increase insulin release, and reduce glucagon release from beta and alpha islet cells in the pancreas respectively, after a meal. In combination, this leads to higher insulin levels and a lowering of blood glucose levels. However, the mechanisms of action of DPP-4 inhibitors are more complex than thought, and may involve the paracrine, endocrine, and neural pathways (Andersen et al., 2018).

A less widely used class of drugs in New Zealand is the alpha-glucosidase inhibitor Acarbose. The mechanisms of action of acarbose involves competitive inhibition of the alpha-glucosidase found in the villi of the small intestine intestinal and thus blocking the enzymatic hydrolysis of oligosaccharides to monosaccharides such as glucose. Acarbose has a similar structure to oligosaccharides and a 10^4^ to 10^5^ times higher affinity for alpha-glucosidase. This means the alpha-glucosidase complexes are competitively inhibited and their availability to oligosaccharides from dietary starch is reduced. Thus, monosaccharide formation decreases and less insulin is required to metabolize glucose, leading to a reduction of food induced increases in blood glucose and insulin (Moelands et al., 2018).

Another class of non-insulin drugs are thiazolidinediones (TZD) and one of these, Pioglitazone, is approved for use in New Zealand. TZD agonistically acts on peroxisome proliferator activated receptors (PPARs) in adipose tissue to enhance insulin action in muscle and liver tissue (Moller, 2001). PPARs are ligand activated transcription factors (nuclear receptor family). Briefly, PPAR activation in adipose tissue leads to activation of adipocyte genes lipoprotein lipase and fatty-acid transporter 1, which lower triglyceride and free fatty acid (FFA) levels, respectively. TNF-α gene expression is also suppressed by PPAR activation. It is known that FFAs and TNF-α are potential mediators of insulin resistance, and also PPAR activation is thought to increase insulin sensitivity. Overall, TZD works by increasing the body's sensitivity to insulin by targeting the PPAR-gamma receptor, which alters the transcription of several genes involved in glucose and lipid metabolism.

Interestingly, two of these classes of drugs were originally derived from natural products. Metformin was discovered from natural products, whose parent compound guanide was originally purified from the plant French lilac Goat's Rue (Koehn and Carter, 2005). Acarbose is a natural microbial product derived from cultures of Actinoplanes strain SE 50 (Goeke et al., 1996).

## Problem With Current T2DM Treatments

TZD and metformin are the two most common classes of drugs used for T2DM treatment worldwide. TZD however are associated with adverse side effects such as gastro-intestinal disturbance, long-term weight gain, fluid retention, and heart failure (Verma et al., 2019). While metformin does not cause weight gain, it does cause gastrointestinal disturbances. Furthermore, it is not considered a complete therapy on its own, as it mostly acts in liver and not muscle tissue. In addition, there are increased risks of cardiovascular disease and kidney diseases associated with DPP-4 inhibitors (Rosenstock et al., 2019). As such, there is a need to find new anti-diabetic agents that can improve insulin resistance, but unlike TZD or insulin, do not induce obesity or other side effects (Feingold, 2000; Heo and Choi, 2019).

Another potential problem is that the use of current T2DM medications is guided by a generic stepwise rubric that does not take into account differences between individuals such as genetic variance. There is growing evidence that there are genetic factors unique to Māori patients that could impact on treatment strategies. A recent genome wide association study identified a variant in the CREBRF gene that is unique to Māori and Pasifika living in New Zealand and that is common in this population (25–30%) but absent in other populations in the world. Surprisingly, although this gene variant is associated with increased BMI, it is also linked to T2DM protection (Krishnan et al., 2018). Together with anecdotal reports that Māori and Pacific respond worse to metformin, this raises the possibility of unique genetic factors regulating responses to medications in Māori and Pasifika. Given that Māori have been using rongoā for hundreds of years with good effect, it is possible there are gene variants or clusters of gene families common among Māori and Pasifika to enable them to process natural rākau rongoā more effectively, as opposed to synthetic drugs like metformin. As such, the need to understand the molecular models of action of anti-diabetic rākau rongoā would be of interest.

## Natural Products as Important Source for Drug Discovery

Natural products as therapeutic medicines have a long history. Due to various adverse side effects of most conventional pharmaceutical drugs, there is a current return in interest of natural products to treat chronic diseases. It is estimated that approximately 25% of all current prescription drugs are plant derived. Furthermore, over the last 25 years of small molecule drug development, 5% were natural products, 27% were natural product derivatives, and 30% were synthetic drugs inspired by natural products (Li et al., 2019; Wright, 2019).

Natural products sourced from plants with fiber and phytochemicals that influence glucose metabolism can be beneficial. Natural herbal supplements that stimulate insulin secretion or utilization, improve insulin binding to its receptor, and improve skeletal muscle function, can be useful in controlling T2DM (Chang et al., 2013; Kooti et al., 2016). Plants are a rich source of phytochemicals that can protect from many chronic diseases such as cancer, diabetes, and cardiovascular disease, which usually target multiple cell signaling pathways (Aggarwal and Shishodia, 2006). The anti-diabetic effect of many natural products from plants have been evaluated and confirmed, which suggest that herbal remedies could represent complementary and alternative treatments, and aid in providing new anti-diabetic agents (Lee et al., 2006; Nistor Baldea et al., 2010).

## Potential for Anti-Diabetic Rākau Rongoā (Māori Herbal Medicine)

New Zealand indigenous flora is considered one of the most unique and diverse in the world, mainly due to geographical isolation (Dawson and Lucas, 2000). Significantly, a high proportion of indigenous flora is endemic to New Zealand, and not found anywhere else on earth. Māori view indigenous flora as taonga (treasure) species, and have strong interests in kaitiakitanga (guardianship) and rangatiratanga (governance), in terms of customary rights and use (New Zealand and Waitangi, 2011a; New Zealand and Waitangi, 2011b).

Many species of indigenous flora have rongoā or medicinal properties that Māori have used for hundreds of years. Rākau rongoā is treasured by Māori as an integral part of Te Wao Nui a Tane (forest mythology), where many are highly prized for its rongoā or medicinal qualities (Williams, 2001) in providing relief for many common ailments and conditions. The mātauranga developed by Māori of the rākau rongoā also holds significant importance. Given the uniqueness and diversity of New Zealand indigenous flora, it is likely that new anti-diabetic treatments will be discovered from these sources. The plant vegetation foods, seeds, roots, nuts, and fruits that formed the basis of traditional Māori diet and rongoā would seem worthwhile targets in a systematic search for anti-diabetic agents. It is also important to understand that Māori believe the beneficial effects of rākau rongoā are not due to the plant alone, but are more importantly due to other traditional influences such as faith in Te Atua God, personal mauri (connection) with Papatūānuku (mother earth), a good sense of oneself as Māori, and a good sense of whakapapa (family history). In Māori communities, natural health and traditional medicinal practices are increasingly widely supported (Williams, 2001).

New Zealand indigenous flora has not been evaluated for their use in the treatment of T2DM. Very little information is available correlating rākau rongoā use and treatment of T2DM disorders. In an effort to determine rākau rongoā with known T2DM efficacy, both traditional and contemporary Māori literature of anti-diabetic rākau rongoā was reviewed. Furthermore, traditional first-hand knowledge sourced directly from kairongoā (rongoā Māori practitioners) were considered and matched with traditional literature. It is also interesting to note that many kairongoā also use rongoā blends or mixtures to help alleviate symptoms of T2DM. Many believe that each plant within the rongoā blend complements each other. Many believe rongoā is not only about plant bioactive compounds having certain medicinal effects on the human physiology, but rongoā is more about supporting and stimulating organs like the liver to heal itself, and for the individual to experience holistic healing at the physical, emotional, mental, and spiritual level.

Early phytochemical studies (Cambie, 1976) also confirm known constituents and the geographical distributions and habitats of known anti-diabetic rākau rongoā throughout Aotearoa New Zealand (New Zealand Plant Conservation Network, 2012). Based on given mātauranga Māori, and earlier phytochemical and geographical studies, the following rākau rongoā have been shown to have anti-diabetic efficacy.

### Karamu (*Coprosma robusta Raoul*)

Karamu (*Coprosma robusta Raoul*) is a large endemic forest shrub with glossy leaves, white flowers, and small dark-orange-red fruit. It can be identified by black stipules and domatia under the leaves. Based on traditional reports, karamu is known to help with diabetes (McGowan, 2018). Furthermore, karamu was taken for bladder inflammation, stomach aches and vomiting (Smith, 1940), and kidney inflammation (Adams, 1945; Williams, 1996; Moon, 2005). Interestingly, early phytochemical studies showed that karamu leaves contained asperuloside (Herissey, 1933).

Karamu is found in both the North and South Island New Zealand (**Table 1**). It is naturalized on the Chatham Islands within a small area between Waitangi and Owenga (New Zealand Plant Conservation Network, 2012). It is common throughout coastal, lowland and lower shrub land montane habitats, and forest open sites.

**Table 1:** Anti-diabetic rākau rongoā with potential rongoā medicinal uses, other traditional uses and geographic location in New Zealand.

Plant - Rākau rongoā	Potential rongoā medicinal uses	Other traditional uses	Geographic location in New Zealand
**Karamu** *Coprosma robusta Raoul*	Stabilise blood sugar levels. Treatments related to inflammation and obesity. Anti-tumour and anti-oxidant properties.	Utilised as food source. Leaves utilised for other rituals. Bark used as a dye.	Found in both North and South Island. Common in coastal and lowland forests, shrublands and open sites including swamps.
**Kūmarahou** *Pomaderris kumeraho A.Cunn*.	Good effect for diabetes. Respiratory (lung) treatment. Skin related treatment - sores, wounds, rashes, skin irritations. Chemopreventative treatment.	Flowers used as a soap. Flowering period - sign for growing food gardens.	Only found in North Island. Coastal to lowland habitats. Found on roadside banks or in shrublands. Prefers full sun and nutrient poor soils.
**Kawakawa** *Macropiper excelsum G.Forst. Miq*.	Used in blends to treat diabetes. A universal rongoā used for a range of ailments.	Utilised as a food source. Used as a head wreath during tangihanga (funeral).	Found in both the North and South Island. More common in North Island. Prefers semi-shaded with free draining and moist soils. Very shade tolerant and frost intolerant.

### Kūmarahou (*Pomaderris kumeraho A.Cunn.*)

Kūmarahou (*Pomaderris kumeraho A.Cunn*.) is an endemic shrub tree, which is identified by its bright yellow flowers in spring September. Traditional reports describe kūmarahou to have good effect for diabetes (Landcare Research, 2020). Furthermore, kūmarahou was used for respiratory lung conditions such as colds, bronchitis, and asthma (Brooker et al., 1981; Moon, 2005). It was commonly known as gum-diggers soap and used for soothing and healing sores, wounds, rashes, and skin irritations (Smith, 1940; Brooker et al., 1981).

Early phytochemical studies showed that kūmarahou leaves contain quercetin, kaempferol, glycosides of quercetin and kaempferol, myricyl acetate, saponins, ellagic acid, certain O-methyl ethers of ellagic acid, leucocyanidin, and leucodelphinidin (Cain and Cambie, 1959).

Kūmarahou only grows on the North Island of New Zealand (**Table 1**). Kūmarahou grows naturally in the upper North Island from Te Paki, down to Kawhia harbour and Te Kuiti in the west and also the northern Bay of Plenty in the east (New Zealand Plant Conservation Network, 2012). Kūmarahou grows within coastal to lowland, in open, early to mid-successional habitats. It is also often found on roadside banks, and in gumland vegetation. It is occasionally seen in forested areas, prefers full sunlight, nutrient poor soils, resents competition, and is prone to phytophora and verticillium wilt.

### Kawakawa (*Macropiper excelsum G.Forst. Miq*.)

Kawakawa (*Macropiper excelsum G.Forst. Miq*.) is a small tree with heart shaped leaves and green-yellow rod shaped berries that is endemic to New Zealand. Rongoā Māori practitioners are known to use kawakawa leaves within rongoā blends to help alleviate symptoms of T2DM (Rawiri-Pukeroa, 2020) such as tiredness, skin infections, and bladder and kidney infections to name of few. Kawakawa is known as a universal rongoā where the leaves and bark were used to treat a range of health conditions. These include nerve pain, tooth infection, toothache, rheumatism, stomach pains, gonorrhea, cuts, wounds, bruises, abrasions, skin disorders, eczema, venereal disease, intestinal worms, boils, abdominal pains, purify blood, bladder complains, kidney troubles, and chest troubles (Brooker et al., 1981; Williams, 1996; Moon, 2005).

Early phytochemical studies conducted on kawakawa leaves showed that diayangambin or Lirioresinol-C dimethyl ether, excelsin, epiexcelsin, and emethoxyexcelsin were all present (Russell and Fenemore, 1973). Furthermore, early studies also showed kawakawa essential oils to contain α-pinene, camphene, 8-phellandrene, n-hexyl acetate, aromadendrene, y-cadinene, myristicin, elemicin, azulene, and palmitic acid (Briggs, 1941). A more recent chemical profiling study on kawakawa revealed significant levels of isovitexin glycoside and vitexin glycoside in hot kawakawa leaf tea, and diayanagambin and elemicin in ethanol extracts, along with low levels of myristicin (Butts et al., 2019).

Kawakawa is found in both the North and South Islands of New Zealand (**Table 1**). Kawakawa is found common from Te Paki, all the way south to Okarito, North Canterbury, and Banks Peninsula (New Zealand Plant Conservation Network, 2012). Kawakawa grows within coastal to lowland and is usually an important understory species in coastal forest. Kawakawa does best in semi-light, within a free draining but permanently moist soil. The plant is very shade tolerant and cold sensitive, and will not tolerate frost.

## Potential Anti-Diabetic Molecular Modes of Action of Selected Flavonoids Known in Karamu, Kūmarahou, and Kawakawa

### Asperuloside

**Figure 1:**
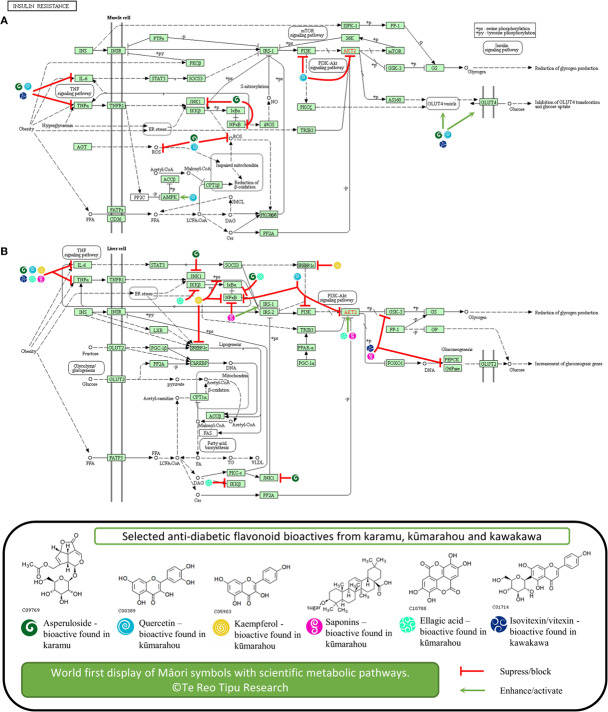
Potential anti-diabetic molecular modes of action of selected flavonoids known in karamu, kūmarahou, and kawakawa in **(A)** liver and **(B)** muscle cell tissue associated with insulin resistance. The figure is original. Copyright permission was granted to use KEGG pathway and compounds (Kanehisa and Goto, 2000).

Karamu contains asperuloside that is a known iridoid glycoside to have anti-diabetic potential (**Figures 1A, B**). A previous study confirmed that chronic asperuloside administration from Eucommia leaf extracts in animal rat models resulted in accelerated fatty acid β-oxidation in the liver and increased GLUT-4 and glucose uptake in skeletal muscles (Fujikawa et al., 2012). Furthermore, asperuloside found within Eucommia extracts were also associated to lower blood glucose levels and increase endogenous anti-oxidant activity in type II diabetic mice (Park et al., 2006).

Accumulating evidence suggests inflammatory processes are involved in the development of pre-diabetes. Asperuloside was also found to significantly downregulate tumor necrosis factor alpha (TNF-α), IL-6, NF-κβ, ERK, and JNK, both *in vitro* and *in vivo* (Qiu et al., 2016; He et al., 2018), a number of pro-inflammatory cytokine markers known to inhibit insulin action (Hotamisligil et al., 1993; Hotamisligil et al., 1994).

### Quercetin

Kūmarahou contains quercetin, a flavonol that has been associated to have therapeutic potential as an anti-diabetic agent (**Figures 1A, B**). The combined effects of quercetin with resveratrol in diabetic rats showed elevated serum blood glucose levels and insulin levels suggesting that co-treatment of quercetin with resveratrol has the potential for use as an alternative therapy for diabetes (Yang and Kang, 2018). Also, a population study with Chinese adults also showed that quercetin intake is inversely associated to T2DM prevalence, suggesting quercetin has preventative activity against T2DM (Yao et al., 2019).

In terms of molecular modes of action, quercetin has been implicated to activate the synthesis and translocation of GLUT-4 glucose transporters and to activate AMPK pathways (Eid et al., 2010). This study showed quercetin from berries to stimulate the AMP-activated protein kinase pathway and enhance basal blood glucose uptake in muscle cells through GLUT-4 translocation, a mechanism similar to metformin treatment for T2DM. Furthermore, animal studies showed that quercetin increased GLUT-4 levels in diabetic rats concluding that quercetin ameliorates hyperglycemia and oxidative stress, by blunting free radical induced toxicity in T2DM (Alam et al., 2014). The anti-diabetic action of quercetin also involved reduction of lipid peroxidation, glucose absorption by GLUT-2, and the inhibition of PI3K in diabetic rats (Coskun et al., 2005; Stewart et al., 2009). Quercetin has also showed to reduce glucokinase activity, hyperglycemia stimulating GLUT-4, hepatic gluconeogenesis, and increased glucose liver uptake (Kobori et al., 2009).

Furthermore, quercetin inhibited activity of tyrosine kinase and the activation of NF-κβ pathway (Dai et al., 2013b). In terms of pre-diabetes, a number of studies have reported the effects of quercetin on TNF-α activity, where quercetin showed to ameliorate TNF-α pro-inflammatory cytokine induced insulin resistance in skeletal muscle tissue (Dai et al., 2013a). Quercetin treated normal rats also showed higher IL-6 and TNF-α levels compared to diabetic rats, implying that quercetin may play an effective role in regulating metabolism in diabetes (Dokumacioglu et al., 2018). Finally, quercetin showed to suppress IL-6, NF-κβ, and PI3K/AKT levels in liver injured mice (Zhu et al., 2017).

### Kaempferol

Kūmarahou also contains kaempferol that is associated to anti-diabetic activity potential (**Figure 1B**). It was found kaempferol ameliorates diabetes in a diabetic animal model, where treatment restored hexokinase activity in the liver and skeletal muscle of diabetic mice; and body weight, calorie intake, body composition, plasma insulin, and glucagon levels were not altered (Alkhalidy et al., 2018). This study suggests that kaempferol may exert anti-diabetic action by promoting glucose metabolism in skeletal muscle and inhibiting gluconeogenesis in the liver. Kaempferol glycoside fractions from Edamame leaves fed to diabetic mice also showed decreased fasting blood glucose and improved insulin resistance, which suggest that kaempferol glycoside fractions improved hyperglycemia and are implicated to having anti-diabetic potential (Zang et al., 2015).

Interestingly, *in vivo* studies also confirmed kaempferol ameliorates insulin resistance through inhibiting pro-inflammatory responses (Luo et al., 2015), particularly by reducing NF-κβ, TNF-α, and IL-6 levels, and restoring insulin resistance by inhibiting phosphorylation of IRS-1. These studies provided evidence that kaempferol may be associated with the reduction of hepatic inflammatory lesions, which is contributing to the improvement of insulin signaling defects in diabetes. Furthermore, kaempferol was found to ameliorate myocardial injury in diabetic rats, by decreasing levels inflammatory markers like TNF-α, IL-6, and NF-κβ (Suchal et al., 2017). Both quercetin and kaempferol from green cocoon of silkworm also showed to reduce blood levels and inhibit TNF-α levels in diabetic mice, along with increased levels of SOD and GSH-px, thus implying diabetic renal protection may occur by reduced oxidative stress mediated by inhibition of TNF-α (Wang et al., 2019).

### Saponins

Kūmarahou contains saponins that are associated to anti-diabetic potential (**Figure 1B**) (Elekofehinti, 2015; El Barky et al., 2017). Interestingly, total saponins from *Stauntonia chinensis* herb administered to diabetic mice revealed it had significant hypoglycemic effect, indicating it may be utilized for T2DM treatment (Xu et al., 2018). Furthermore, a preliminary study on the mechanisms of total saponins from *Entada phaseoloides* against diabetes was also conducted (Zheng et al., 2012b).

Interestingly, the effects of total saponins from various natural sources, showed to ameliorate diabetes through inhibiting pro-inflammatory responses showed by reduced TNF-α levels (Liu et al., 2012; Zheng et al., 2012a; Yu et al., 2015). Furthermore, total saponins from *Dioscorea nipponica*, down regulated NF-κβ and up-regulate protein expression of IRS-1, GLUT-4, p-AKT, and p-AMPK; and decreased gene expression of TNF-α, IL-6, PEPCK, and G6Pase and GSK-3 in liver (Yu et al., 2015), implicating the plant extracts potential for pre-diabetic and diabetes treatment.

### Ellagic Acid

Kūmarahou contains ellagic acid that is also been shown to attenuate hyperglycemia potential (**Figure 1B**). Studies of diabetic rats treated with ellagic acid revealed a decrease blood glucose levels confirming the anti-hyperglycemic potential of ellagic acid (Farbood et al., 2019). Furthermore, ellagic acid found in *Emblica officinalis* was also implicated to anti-diabetic activity in pancreatic β-cells, *in vitro* and *in vivo* (Fatima et al., 2017). Ellagic acid was also found to alleviate insulin resistance in diabetic female rats to understand its beneficial effects for the treatment of hepatic complications in T2DM (Polce et al., 2018). This study further showed that ellagic acid treatment activated insulin signaling in the liver, by increasing levels of phosphorylated AKT.

Interestingly, early *in vivo* studies showed ellagic acid to significantly reduce serum levels of pro-inflammatory cytokines, interleukins IL-1β, IL-6, and TNF-α (Chao et al., 2010; Ahad et al., 2014). Ellagic acid also showed to decrease blood glucose and TNFα levels, and inhibit up-regulation of NF-κβ and IKK-β in a diabetic nephropathy mouse model, suggesting that ellagic acid ameliorates diabetic induced oxidative injury (Zhou et al., 2019).

### Leucocyanidin, Leucodelphinidin, and Derivatives

Kūmarahou contains leucocyanidin, leucodelphinidin, and derivatives, which are also linked to anti-diabetic activity. Interestingly, a leucocyanidin derivative from the bark of *Ficus bengalensis* demonstrated anti-diabetic action, *in vivo* (Kumar and Augusti, 1989). Leucocyanidin was also putatively isolated from plantain banana and showed to possibly have anti-diabetic effect, *in vivo* (Kumar et al., 2013). Earlier *in vivo* studies also showed a leucodelphinidin derivative isolated from the bark of *Ficus bengalensis* demonstrated hypoglycemic action both in normal and diabetic rats (Geetha et al., 1994).

### Isovitexin and Vitexin Glycosides

Kawakawa contains significant levels of isovitexin and vitexin glycosides that are associated to anti-diabetic potential (**Figures 1A, B**). The administration of isovitexin and vitexin in diabetic rats reduced blood glucose and also showed to exhibit *in vivo* alpha-glucosidase inhibition (Choo et al., 2012). Furthermore, extracts of *Ficus deltoidea* which contains enriched levels of isovitexin and vitexin, also inhibited hyperglycemia in diabetic rats, concluding that the herb can be used to alleviate diabetic complications (Farsi et al., 2014). The *Ficus deltoidea* extracts down-regulated PEPCK and G6Pase expression, yet up-regulated GK and PPARγ gene expression in liver. Further, *Ficus deltoidea* extracts increased expression of GLUT-4 expression in muscle skeletal, leading to normalize hyperglycemia. The Isovitexin and vitexin in bamboo leaf extracts was also shown to inhibit α-amylase, by interacting with its active site through the glucoside linking with the 3'-hydroxyl group of the active site and retarding digestion of starch (Yang et al., 2014). The study showed potential of the bamboo extracts as a starch-based additive to adjust postprandial hyperglycemia.

Isovitexin found in *Gentiana lutea* plant has also been implicated with endothelial inflammation in diabetic rats, where pro-inflammatory cytokine TNF-α production was blocked by isovitexin treatment (Kesavan et al., 2016), showing possible aide in diabetic cardiomyopathy. As such, it is possible that isovitexin which is found in kawakawa leaf extracts at significant levels, may also inhibit TNF-α production in injured endothelial tissue related to diabetic cardiomyopathy. Lastly, *Cynometra cauliflora* leaves containing major compound vitexin were tested in high-fat diet mice, showing decreased serum levels of TNF-α and IL-6 (Seyedan et al., 2019), and up-regulation of GLUT-4 and IRS-1 in white adipose tissue.

## Challenge of Clinically Proven Rākau Rongoā

Many of the claimed efficacies of rākau rongoā with anti-diabetic properties are not based on robust scientific laboratory based testing and human clinical trials of a modern standard, and the mode of action is not known or scientifically proven. As such, the major challenge remains in the identification and validation of active anti-diabetic rākau rongoā extracts to support its efficacy for T2DM.

Furthermore, not only do Māori use rākau rongoā for treating diagnosed disease but many use it as a regular general tonic. In this context it has the potential to combat the metabolic impairments that are seen in the pre-diabetic state and so potentially delay the development of full T2DM. However, no pre-clinical or clinical research has yet been done on this aspect of rākau rongoā use.

Interestingly, the C-reactive protein or CRP, is a major acute protein considered for low grade systematic inflammation in chronic disease like cancer, cardiovascular (Ridker, 2003) and T2DM (Hu et al., 2004). Previous studies have reported associations of CRP with insulin resistance and pre-clinical T2DM (Hotamisligil, 2010). As mentioned earlier, pro-inflammatory cytokines such as IL-6 and TNF-α are implicated to promote insulin resistance by interfering with the insulin signaling pathway, leading to the pre-clinical manifestation of T2DM (Gregor and Hotamisligil, 2011).

Mammalian cell culture systems modeling the major tissues involved in glucose metabolism (i.e. liver, muscle, fat, and the insulin producing pancreatic beta-cells) provide an important *in vitro* scientific model in which initial studies of rākau rongoā could be undertaken. These provide very important first line evidence of efficacy and mechanistic impacts.

The evaluation of *in vivo* efficacy is best tested in animal model systems as this enables testing of effects on the integrated physiological responses. Animal models include diet induced and genetic models of obesity of relevance to T2DM (Al-awar et al., 2016) and the streptozotocin (STZ) induced diabetic model used to study impacts on T1DM efficacy (Gvazava et al., 2018). These are thought to be of more direct relevance to therapeutic outcomes for metabolic disease in humans. Therefore, animal models are regarded as pre-clinical research and are widely used in the search and validation of new treatments for metabolic disease.

## Social Determinant of Indigenous Health: Language, Culture, Traditional Knowledge, and Identity - Mātauranga Māori - Heal The Land, Heal the People

There has been growing recognition that “health” is more than an individualistic, biomedical concept; health is also determined by social circumstances and contexts (Lines et al., 2019). These social determinants of health involve the conditions under which people live and work, and include diverse factors such as language, culture, and identity. Indigenous culture is a dynamic and adaptive system of meaning that is learned, shared, and transmitted from one generation to the next and is reflected in the values, norms, practices, symbols, ways of life, and other social interactions of a given culture (Kreuter and McClure, 2004). Relationships, interconnectivity, and community are fundamental to these dynamics (Lines et al., 2019).

For Māori, the indigenous peoples of New Zealand, there is an intrinsic connection between the health of the people and the health of their land (McGowan, 2017). Māori developed mātauranga of their whenua over centuries, which was passed down from their ancestors who originated from Hawaiiki (Smith, 1898). As such, mātauranga Māori is about connection to Papatūānuku or whenua land (McGowan, 2017). Once those connections are broken, mātauranga Māori becomes less of a living knowledge. A disconnection of mātauranga Māori commonly occurs when it is taken out of context in which it originated. Mātauranga Māori is about places and people connected to those places. Mātauranga Māori is much more than traditional information, knowledge, facts, figures, and data used in proposals and strategies, with little thought of what it means to Māori, the people who developed that knowledge in the first instance (McGowan, 2017). It is the Māori way of knowing and keeping knowledge alive, which is passed on to the next generation. Mātauranga Māori is a knowledge of the land from the people who belong to the land. It is the result of their interaction with the whenua over many generations. It is the knowledge they have accumulated by living on the land, working with the land, harvesting from the land, all the time listening, watching, and caring to ensure that they can continue to survive. Mātauranga Māori is not an artefact, but is a living knowledge that is being constantly enriched and extended in people's lives as the world continues to change. It is about survival and retaining those connections to the whenua by keeping alive the stories of the land. Mātauranga Māori is about the future, where it is founded on the past and focused on the survival for future generations (McGowan, 2017).

Preserving mātauranga of the medicinal properties of rākau rongoā has proven to be a major challenge and concern among many Māori. Although much work has been done to safeguard mātauranga Māori, the issue still has not been adequately resolved. In an effort to resolve this issue, recent discussion among Māori scientists and researchers have raised the need for an Independent Mātauranga Māori Commission to be developed, to take the process off the New Zealand Crown and place it back into the hands of Māori (Māngai, 2020).

## Māori View to Animal Testing of Rākau Rongoā

Māori believe that all people, plants, and animals are descendants of Ranginui (father sky) and Papatūānuku (mother earth). Māori have been using rākau rongoā to treat and alleviate disease and health issues for hundreds of years, where some see no point to provide pre-clinical evidence in testing rongoā on animals because they know it works. However, these views represent the minority.

Many Māori support the use of animal testing to understand the effects of rongoā at the physiological and molecular level, if that knowledge is unknown. Many Māori support animal testing of rākau rongoā if the research is conducted under the guidance and protection of a Māori kaumatua (elder), kairongoā (rongoā Māori practitioner), and Māori kairangahau (researcher). As mentioned earlier, Māori have strong interests in kaitiakitanga and rangatiratanga, and support animal testing of rākau rongoā if it is preserved and governed under their guidance.

Māori view the intake of rongoā by animals as a very natural process, which can help guide laboratory research if conducted in a culturally humane and safe environment for the animal and rākau rongoā under investigation. Furthermore, it is important that a karakia (prayer) is given by a Māori kaumatua before the research commences and ends, including when the animal is euthanized humanely.

## Rongoā Māori Mana Motuhake (Self-Determination for Traditional Māori Healing Practices)

Mainstream health systems are constantly charging Māori to validate the efficacy of their rongoā Māori practice based on mainstream health systems, without recognizing that Māori have their own body of knowledge and practice systems based on mātauranga Māori and tikanga Māori (traditional kaupapa Māori protocol) (Koia, 2016). This is viewed as institutional racism and Crown inaction on health equity in New Zealand (Came et al., 2019). Furthermore, this also supports historical practices of colonization and forced assimilation enacted by the Crown as profoundly racist (Smith, 2012). Furthermore, colonial policies informed by superior Pākehā people, institutions, and systems, have allowed entitlement of Pākehā to resources and power, including those related to traditional rongoā Māori practices. As such, the New Zealand Crown are thought to be in breach of Treaty of Waitangi obligations in terms of inequity between mainstream health systems and traditional rongoā Māori healing practices.

In relation to rongoā, the Waitangi tribunal report Wai262 (Waitangi Tribunal, 2011) found that mātauranga Māori of rākau rongoā is created by Māori. The report states that Māori believe that mātauranga Māori of rākau rongoā should not be exclusively owned, as it would be wrong to exclude others from experiencing the wealth and richness of Te Ao Māori. However, Māori object to individuals who seek to exploit that knowledge for commercial gain without proper acknowledgment of the prior rights to kaitiaki, given that the relevant mātauranga Māori will always be a creation of Māori.

The work conducted by kairongoā Māori practitioners in providing alternative health therapies, has too often been ignored due to the lack of scientific and clinical research to verify their practice. As such, their work is thought to have no value in terms of meeting present day health needs, and therefore warrants no support to enable healers to bring rākau rongoā to those they are able to help. This in turn contributes to loss of mātauranga Māori, which is the basis of rongoā Māori (McGowan, 2017). A major objection raised by mainstream health providers, is the lack of scientific and clinical research to support the use of rākau rongoā, which therefore creates resistance towards the acceptance of rākau rongoā in the community.

The challenge lies to provide clinically proven anti-diabetic rongoā extracts to supports its efficacy for T2DM mate huka, under a kaupapa Māori framework and in the hands of kaupapa Māori researchers with biomedical and molecular experience who advocate the importance of rongoā Māori mana motuhake. The Wai 262 report (Waitangi Tribunal, 2011) supports the emergence of mātauranga Māori from the domination of Pākehā knowledge system, to flourish once again in Māori hands.

The Wai 262 report also states that health is a key area, and the protection and enhancement of rongoā are prominent. The report seeks not to replace Western medicine, but rather to ensure the benefits of rongoā can be enjoyed as a complement (Waitangi Tribunal, 2011).

As the gap between Māori and non-Māori with T2DM is significant, cultural approaches to T2DM care for Māori ought to be considered. Given that T2DM is most prevalent among Māori, clinically proven rākau rongoā with anti-diabetic potential could be utilized in the prevention of T2DM or management of T2DM and other related complications once they arise.

Further, there is a need for Māori to re-engage with their cultural roots that once guided them toward having good health and in strengthening the connection they have with their whenua land, their whakapapa family history and with themselves to restore balance in their journey of healing and good health.

## Te Reo Tipu Research: Kaupapa Māori Molecular and Genomic Research of Taonga Flora

Based on traditional reports and knowledge, karamu, kūmarahou, and kawakawa each display anti-diabetic potential. Remarkably, no molecular or biomedical research has been conducted to confirm the anti-diabetic efficacy of these rākau rongoā and to understand the mechanisms by which these effects are achieved. Although early phytochemical studies confirm known constituents, research is yet to be performed to validate anti-diabetic agents of the given rākau rongoā. 2D cell culture and animal model systems provide ways to study the effectiveness of anti-diabetic agents sourced from rākau rongoā.

**Figure 2:**
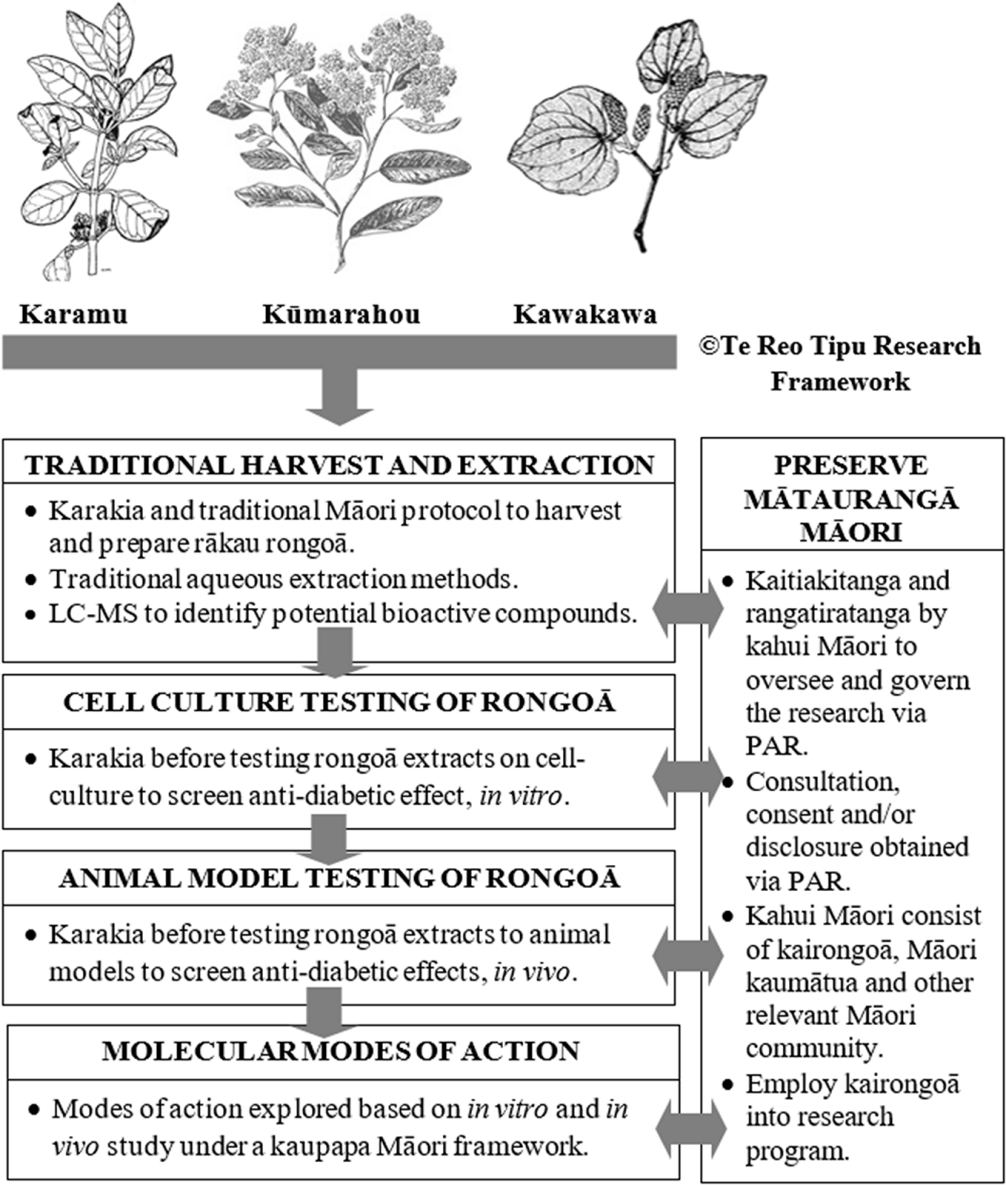
Te Reo Tipu Research - Kaupapa Māori molecular research scheme to study clinically proven karamu, kūmarahou and kawakawa rākau rongoā used for T2DM ‘mate huka’. This figure is original. The karamu, kūmarahou and kawakawa images were obtained from Te Ara: The Encyclopedia of New Zealand, which is licensed by Manatū Taonga Ministry for Culture and Heritage for re-use under the Creative Commons Attribution-Non Commercial 3.0 New Zealand Licence.

The preparation of rongoā from these should be performed following certain principles and **Figure 2** illustrates a kaupapa Māori molecular research scheme to undertake pre-clinical and clinical studies to test efficacy of karamu, kūmarahou, and kawakawa rākau rongoā in T2DM “mate huka.” Harvesting and aqueous extraction of rākau rongoā ought to be performed under the direction of a kairongoā or Māori kaumātua. In line with traditional Māori protocol, karakia is essential to acknowledge and thank the gift of Tane Mahuta prior to harvesting any rākau rongoā plant material. Harvesting rākau rongoā involves considering the needs of others, ensuring sustainability in the forest, being gentle with footprints in the forest, harvesting the eastside of the plant by hand, never harvest in the rain and to harvest leaves during growing season (Kerridge, 2014).

Human cell lines used in cell culture research are derived from individuals who have given consent for their cells to be used for research purposes, prior to passing. In line with tikanga Māori, it is believed that once these cells are handled by a researcher, both the researcher and laboratory space enter into a state of tapu or sacredness. Therefore, karakia ought to be offered by a Māori kaumātua before any cell culture screening is performed to ensure all personnel are kept safe and more importantly to acknowledge the deceased person who donated their cells for research purposes (**Figure 2**). Pre-clinical animal testing of rākau rongoā ought to be conducted in an environment that is culturally sensitive and respectful to Māori and under the guidance of a kaumātua and kairongoā.

Participatory action research (PAR) approaches related to health programs has allowed partnerships between researchers and community members to reflect, collect data, and action aims to improve health and reduce health inequities through involving the people who, in turn, take actions to improve their own health. As such, PAR methods as shown in **Figure 2** will allow effective partnerships between molecular researchers and relevant Māori community members such as kairongoā and kaumātua elders to reflect, collect data and action aims in an effort to improve T2DM “mate huka” within the Māori community. This review also suggests a framework that can be used by molecular researchers to employ kairongoā and kaumātua into their research programs.

In conclusion, kaupapa Māori molecular cell research on anti-diabetic rākau rongoā has the potential to (1) provide the first phase of screening and testing anti-diabetic agents found in rākau rongoā with known anti-diabetic efficacy through 2D cell-culture assay approaches, and (2) give pre-clinical evidence for anti-diabetic properties of rākau rongoā plant extracts using animal model systems. It also has the potential to (3) identify new anti-diabetic agents from rākau rongoā, which may lead to clinically targeted outcomes, and (4) contribute towards the development of safe natural supplementary products that could be used in diabetes management plan of Māori and non-Māori with T2DM “mate huka,” thus possibly impacting the provision of health services for all New Zealanders. Lastly, it has the potential to (5) develop a culturally relevant approach to T2DM “mate huka” care among Māori.

## Author Contributions

JK conceptualized and wrote the article. PS significantly contributed to the design, drafting, and revision of the work for intellectual content.

## Funding

This review was funded through the Health Research Council of New Zealand. HRC Ref ID 17/487. Research title Te Reo Tipu - a bittersweet quest for new anti-diabetic agents in rongoā rākau.

## Conflict of Interest

The authors declare that the research was conducted in the absence of any commercial or financial relationships that could be construed as a potential conflict of interest.

## Glossary of Māori Terms

**Table:**

Māori term	Meaning
Kairongoā	Rongoā Maori practitioner.
Kaitiaki	Guardian, custodian, caregiver, keeper, and steward.
Kaitiakitanga	Guardianship.
Karakia	To recite a prayer.
Karamu	*Coprosma robusta Raoul*.
Kaumātua	Māori elder (man/woman). A person of status within the family.
Kaupapa Māori	Māori theme, proposal, issue, or topic.
Kawakawa	*Macropiper excelsum G.Forst. Miq*.
Kūmarahou	*Pomaderris kumeraho A.Cunn*.
Māori	First nations and indigenous Peoples of New Zealand.
Māori kairangahau	Māori researcher.
Māori mana motuhake	Māori movement of self-determination.
Mātauranga Māori	Traditional Māori knowledge.
Mate huka	Literal translation “mate = disease, huka = sugar.” Māori terms used to describe type II diabetes.
Mauri	Life principle to describe life force and source of emotion and connection.
Pākehā	New Zealander of European descent.
Papatūānuku	Earth mother and wife of Ranginui. All living things originate from them.
Pūtaiao	Science.
Rangatiratanga	Governance, domination, rule, control, and power.
Ranginui	God of the sky and husband of Papatūānuku. All living things originate from them.
Rongoā	To remedy, treat, heal, cure, and apply medicine.
Rongoā Māori	Natural remedy of holistic traditional Māori healing practice.
Rongoā Māori mana motuhake	Self-determination for traditional Māori healing practices.
Rākau rongoā	Māori herbal medicine.
Rongoā wairākau	Decoction made from boiling Māori herbal medicine in water.
Tane Mahuta	Māori God of the forests and birds. One of the children of Ranginui and Papatūānuku.
Tangihanga	Funeral. One of the most important gatherings in Māori society with strong cultural protocols.
Taonga	Treasure and anything prized.
Te Ao Māori	The Māori worldview.
Te Atua	God
Te Reo Tipu	A kaupapa Māori framework for molecular and genomic research on taonga flora. Literal translation “The voice of the plants.”
Te Wao Nui a Tane	Forest Māori mythology.
Tikanga Māori	Māori customary system of values and practices.
Tohunga	A chosen healer.
Whakapapa	Family history or geneology.
Whenua	Land.
